# Physiology-guided volume optimization in patients with acute exacerbations of heart failure complicated by various comorbidities: two case reports

**DOI:** 10.3389/fcvm.2026.1877673

**Published:** 2026-09-08

**Authors:** Hua Bai, Dahai Bing, Shujuan Wang, Jiajia Li, Peng Liu, Zepeng Ren, Xi Liu

**Affiliations:** 1 Affiliated Ordos Clinical College of Inner Mongolia Medical University, Ordos City, Inner Mongolia, China; 2 Department of Cardiovascular Medicine, Ordos Central Hospital, Ordos, Inner Mongolia, China; 3 Department of Cardiovascular Medicine, Second Affiliated Hospital of Harbin Medical University, Harbin City, Heilongjiang, China

**Keywords:** dilated cardiomyopathy, heart failure, renal insufficiency, sepsis, volume management

## Abstract

**Background:**

Traditional volume management for acute decompensated heart failure emphasizes strict fluid restriction. However, this may be harmful in patients with relative hypovolemia (e.g., poor intake or sepsis). We report two cases managed with a physiology-guided, individualized maintenance fluid strategy (avoiding routine restriction) and present them as hypothesis-generating observations.

**Case presentation:**

Case 1: A 69-year-old man with dilated cardiomyopathy presented with acute exacerbation, hypotension, prerenal azotemia (urea/creatinine >20:1), and anorexia. We used maintenance fluids (approximately 35 mL/kg/day as an initial empirical target, adjusted clinically) while continuing guideline-directed medical therapy (GDMT). Symptoms improved; NT-proBNP fell from 20,470 to 2,669 pg/mL. Case 2: An 83-year-old woman with sepsis from an infected pressure ulcer developed acute heart failure. We used a similar maintenance fluid strategy (not aggressive resuscitation) plus antibiotics and gradual GDMT. Infection resolved, NT-proBNP fell from 14,995 to 365 pg/mL.

**Conclusion:**

In selected patients with ADHF and possible hypovolemic factors, a physiology-guided maintenance strategy may be considered as an alternative to strict restriction. This strategy may help interrupt the hypothesized vicious cycle of hypoperfusion and renal injury, and is compatible with the optimization of GDMT. However, causality cannot be inferred because multiple interventions were given simultaneously. These observations are hypothesis-generating and require prospective validation. Individualized management is essential.

## Introduction

Volume management is a cornerstone of heart failure treatment, particularly during the acute decompensated phase. For decades, clinical guidelines have recommended that patients with heart failure limit their sodium intake (<2 g/day) and fluid intake (1.5–2.0 L/day) to reduce sodium and water retention and alleviate symptoms of congestion (1, 2). This strategy is based on the pathophysiological understanding that heart failure is a state of sodium and water retention (10).

However, a growing body of evidence challenges routine fluid restriction strategies. Several randomized controlled trials and meta-analyses have failed to demonstrate that fluid restriction improves clinical outcomes in patients with heart failure (3–5) The 2024 clinical consensus statement from the European Society of Cardiology Heart Failure Association notes that there is currently a lack of high-quality evidence supporting routine salt and fluid restriction in patients with acute or chronic heart failure; instead, such restrictions may lead to adverse effects such as malnutrition, thirst, reduced quality of life, and abnormal neurohormonal activation (6, 7).

In clinical practice, inadequate fluid intake due to gastrointestinal congestion or comorbidities can lead to a vicious cycle: reduced effective circulating volume → prerenal azotemia → neurohormonal activation → worsening heart failure. In this context, strict fluid restriction may exacerbate rather than alleviate the problem.

The dilemma is particularly pronounced in heart failure with sepsis: the Surviving Sepsis Campaign recommends fluid resuscitation (30 mL/kg), while heart failure guidelines emphasize restriction. Evidence for managing this conflict is scarce.

Notably, objective bedside tools including inferior vena cava (IVC) ultrasound, lung ultrasound and bioelectrical impedance analysis (BIA) are well-established complementary modalities to quantify intravascular volume status, distinguish relative hypovolemia from pure congestion, and predict decompensation events (1–3). Nevertheless, these objective examinations were not performed in the present two cases, which represents a critical limitation of our clinical assessment.

This article reports two cases of acute heart failure managed using a physiology-guided volume optimization strategy (i.e., avoiding routine restriction while providing fluids to meet estimated maintenance requirements, not aggressive resuscitation). By detailing the course and outcomes, we explore the hypothesis-generating rationale and limitations of this approach.

## Case presentation

### Case 1: acute exacerbation of chronic heart failure with prerenal azotemia

#### Patient information and chief complaint

A 69-year-old man was admitted on May 26, 2024, presenting with “shortness of breath on exertion for 16 years, worsening over the past 2 days.” The patient first experienced shortness of breath on exertion in 2008 and was diagnosed with dilated cardiomyopathy at the First Affiliated Hospital of Sun Yat-sen University between 2011 and 2013. Since 2013, he has been under long-term, regular follow-up and medication at our hospital, with good compliance. Two days ago, following a cold, his dyspnea worsened; he now experiences shortness of breath even at rest, accompanied by marked loss of appetite, and subsequently developed symptomatic hypotension.

#### Past medical history

History of type 2 diabetes for over 10 years and gout for 5 years. Denies history of hypertension. History of smoking and alcohol consumption; both have been discontinued. Multiple family members have a history of dilated cardiomyopathy. Long-term Medication Regimen: Sacubitril/valsartan (200 mg bid), metoprolol sustained-release tablets (190 mg qd), dapagliflozin (10 mg qd), finerenone (20 mg qd), vildagliptin (10 mg qd), and torasemide (20 mg qd or bid).

#### Physical examination

Pulse 100/min, respiratory rate 20/min, blood pressure 96/60 mmHg. Jugular venous distension present. Skin turgor was reduced and oral mucous membranes were dry, suggesting relative hypovolemia. Slight bibasal crackles. Heart sounds muffled. No lower limb edema.

#### Supportive tests

NT-proBNP 20,470 pg/mL; urea 18.50 mmol/L, creatinine 203.0 μmol/L, urea/creatinine ratio 91.1 (>20:1) – prerenal pattern. IL-6 16.80 pg/mL, procalcitonin 17.90 ng/mL. Chest x-ray: cardiomegaly. ECG: sinus rhythm, LBBB (QRS 142 ms). Echocardiography: LVEF 40%, left ventricular end-diastolic diameter 69 mm, moderate-to-severe mitral regurgitation. Cardiac MRI: LVEF 16.33%, consistent with dilated cardiomyopathy. The discrepancy between echo (40%) and MRI (16.33%) is explained by inter-modality variability (MRI gold standard), different loading conditions (hypovolemia and tachycardia may artificially increase echo-derived LVEF), and suboptimal acoustic windows. The MRI result is considered more accurate for true systolic function. The discrepancy between echocardiographic LVEF (40%) and cardiac MRI (16.33%) warrants explanation. First, echocardiography (biplane Simpson method) and cardiac MRI use different volumetric algorithms, with MRI being the gold standard for accurate LVEF assessment. Second, the echocardiogram was performed during a state of relative hypovolemia and tachycardia, which may have reduced left ventricular volumes and artificially increased fractional shortening, thereby overestimating LVEF. Third, suboptimal acoustic windows due to the patient's body habitus may have introduced foreshortening artifact. For clinical decision-making, we relied primarily on the MRI-derived LVEF (16.33%) for prognostic assessment and for the eventual LVAD referral, as MRI provides more accurate and reproducible volumetric data. However, the echocardiographic finding of 40% was not ignored; it indicated that systolic function was not uniformly and irreversibly depressed, which supported our decision to continue and optimize GDMT while stabilizing the patient with the maintenance fluid strategy.

#### Clinical presentation and treatment approach

The patient had both pulmonary congestion (crackles, high NT-proBNP) and prerenal azotemia (low BP, high urea/creatinine, poor intake, dry mucosa). Traditional therapy (more diuretics + fluid restriction) risked worsening renal function. We therefore chose a physiology-guided maintenance fluid strategy: restore effective circulating volume while continuing low-dose diuretics (torasemide 20 mg/day). Predefined safety triggers to slow/stop fluids included: increasing crackles, worsening dyspnea or SpO₂ < 90%, rising JVP, weight gain >1 kg/day, or need for diuretic escalation. Reassessment was done every 4-6 h.

#### Interventions and clinical course

Volume replacement: Oral intake encouraged. Intravenous fluids (glucose/electrolytes) were given at an initial empirical target of approximately 35 mL/kg/day. This target was derived from the Holliday-Segar maintenance fluid formula, which estimates daily fluid requirements for euvolemic adults at approximately 30–40 mL/kg/day (Holliday MA, Segar WE. The maintenance need for water in parenteral fluid therapy. Pediatrics. 1957;19(5):823–832). We selected 35 mL/kg/day as a mid-range starting point for this patient because of clear signs of relative hypovolemia (prerenal azotemia, dry mucous membranes, hypotension) and to avoid both aggressive resuscitation and strict restriction. This target is not evidence-based for heart failure and was adjusted daily based on clinical reassessment (crackles, JVP, dyspnea). Precise oral intake and daily weights were not consistently recorded; this is a limitation.

Rationale for crystalloid rather than colloid volume expansion: Colloid agents can produce faster and longer-lasting intravascular volume expansion compared with crystalloids, which theoretically benefits patients with severe relative hypovolemia4. However, high-quality meta-analyses and Cochrane reviews have failed to confirm a mortality benefit of colloids over balanced crystalloids in critically ill populations4–5. For this patient with systolic heart dysfunction and baseline congestion risk, colloids carry additional risks including prolonged volume overload, worsening renal filtration burden, and increased bleeding risk from albumin formulations. Balanced glucose-electrolyte crystalloids offer steady, controllable fluid delivery, allowing real-time downward adjustment at the first sign of pulmonary congestion, which aligned better with our individualized volume optimization goal.

**Figure 1:**
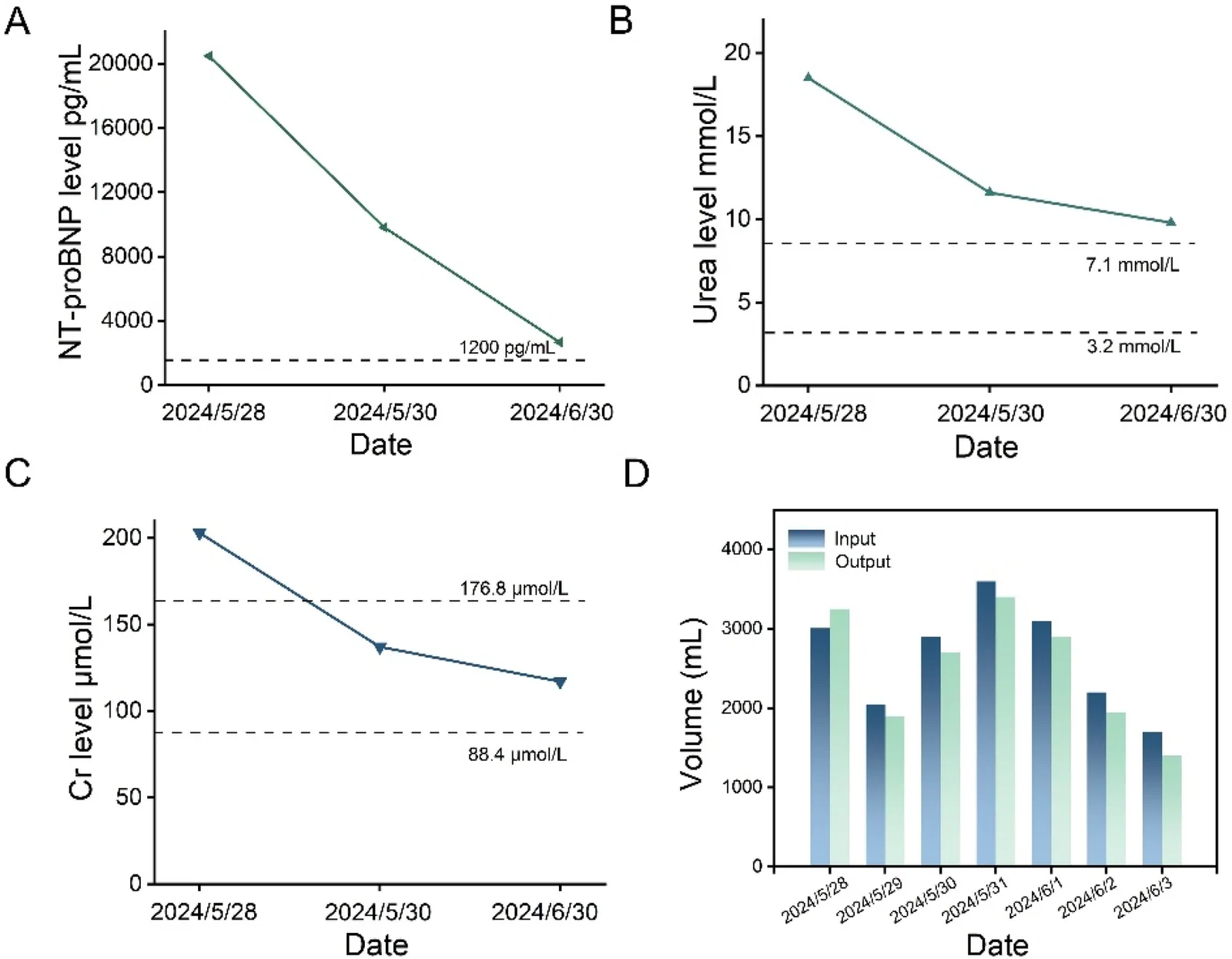
**(A)** NT-proBNP, **(B)** creatinine, **(C)** urea over time (admission and day 30). **(D)** Daily fluid balance for the first 7 days. The histogram clearly demonstrates a consistent negative fluid balance within the first 24–48 h after admission, matching the hourly urine output monitoring data recorded clinically. Objective volume assessment [e.g., IVC ultrasound, lung ultrasound, bioelectrical impedance analysis (BIA)] was not performed, and daily weights were not consistently recorded—these are major limitations, as BIA and IVC ultrasound are validated tools to differentiate intravascular hypovolemia and tissue fluid overload.

Early urine output monitoring: Hourly urine volume was documented for the first 48 h of admission, in accordance with ESC heart failure diuretic guidance and inpatient heart failure protocols6–7. Within the initial 24 h, cumulative urine output reached 1,820 mL, with hourly urine output consistently maintained above 150 mL/h after low-dose torasemide administration; a sustained negative fluid balance was achieved during the critical early treatment window (Figure 1D). Serial urine output surveillance helped avoid delayed identification of inadequate decongestion response.

Diuretic strategy: Torasemide 20 mg/day (no increase).

GDMT: Continued sacubitril/valsartan, metoprolol, dapagliflozin, finerenone. Ivabradine added for ventricular arrhythmias.

#### Outcome

Symptoms improved rapidly. Day 30 follow-up: NT-proBNP 2,669 pg/mL (87% ↓), creatinine 137 μmol/L, inflammatory markers normal. The maintenance fluid strategy served as a bridge to LVAD implantation by stabilizing hemodynamics and preserving renal function; however, its independent contribution to outcome cannot be isolated from concurrent GDMT and supportive care. Following stabilization, the patient was transferred to Fuwai Hospital (Beijing) for further evaluation and subsequently underwent successful Left Ventricular Assist Device (LVAD) implantation. According to follow-up information, his postoperative recovery has been satisfactory, and he remains in stable condition. However, we do not have access to the specific details of his post-implantation management at the receiving hospital (Figure 1).

### Case 2: sepsis complicated by acute left heart failure

#### Patient information and chief complaint

An 83-year-old woman was admitted on December 30, 2024, presenting with “a pressure ulcer for 2 months, and chills accompanied by dyspnea and slurred speech for 2 days.” Two months ago, the patient sustained a fracture of the anterior wall of the left acetabulum and the inferior ramus of the pubis following a fall. Due to prolonged bed rest, she developed a Stage IV pressure ulcer in the sacrococcygeal region (approximately 3 cm × 5 cm, with purulent exudate on the surface). Two days ago, she experienced recurrent chills and progressively worsening dyspnea. Initially diagnosed with “cerebral infarction” in the emergency department and admitted to the Department of Neurology, the patient was subsequently transferred to the Department of Cardiology due to clear signs of heart failure.

#### Past medical history

History of hypertension for 10 years, treated with oral irbesartan and hydrochlorothiazide; chronic bronchitis for over 1 year; lacunar cerebral infarction for over 1 year.

#### Physical examination

Pulse 124 beats/min, respiratory rate 25 breaths/min, blood pressure 91/45 mmHg. General condition poor; jugular vein distension. Wet rales at the lung bases bilaterally. Heart sounds muffled; S1 variable in intensity; markedly irregular rhythm; heart rate 135 beats/min. Moderate edema of both lower extremities.

#### Laboratory findings

Laboratory tests on admission: white blood cells 12.44 × 10⁹/L, platelets 36 × 10⁹/L (suggesting infection-related); NT-proBNP 14,995 pg/mL; creatinine 164 μmol/L, urea 21 mmol/L. SOFA score: 6 (an increase of ≥2 points from baseline), meeting the criteria for sepsis. Blood cultures and pressure ulcer exudate cultures both identified Micrococcus (anaerobic G + cocci). ECG: Rapid atrial fibrillation. Bedside echocardiography: LVEF 45%, mild reduction in left ventricular systolic function.

#### Clinical presentation and treatment approach

The patient had both sepsis (SOFA 6) and acute heart failure—a classic volume management conflict. Instead of aggressive resuscitation or strict restriction, we used a physiology-guided volume optimization strategy (approximately 35 mL/kg/day as initial empirical target, adjusted clinically), combined with antibiotics and gradual GDMT. We emphasize that the favorable outcome cannot be attributed solely to the fluid strategy. Conflict reconciliation between sepsis resuscitation and heart failure decongestion: The 2021 Surviving Sepsis Campaign international guidelines recommend initial 30 mL/kg crystalloid bolus resuscitation as first-line therapy for septic patients (8). However, this aggressive volume loading carries high risks of acute pulmonary edema for patients with pre-existing left ventricular systolic dysfunction, as supported by network meta-analysis data of patients complicated with both severe infection and heart failure (9). For this elderly patient with baseline heart failure, elevated NT-proBNP and bilateral pulmonary rales, a moderate maintenance crystalloid regimen (35 mL/kg/day glucose-saline balanced solution) was selected as a compromise: the regimen avoided insufficient organ perfusion caused by excessive fluid restriction, while preventing the massive fluid overload induced by standard sepsis bolus resuscitation. Colloid formulations were not adopted here for identical safety concerns described in Case 1.

#### Interventions and clinical course

**Figure 2:**
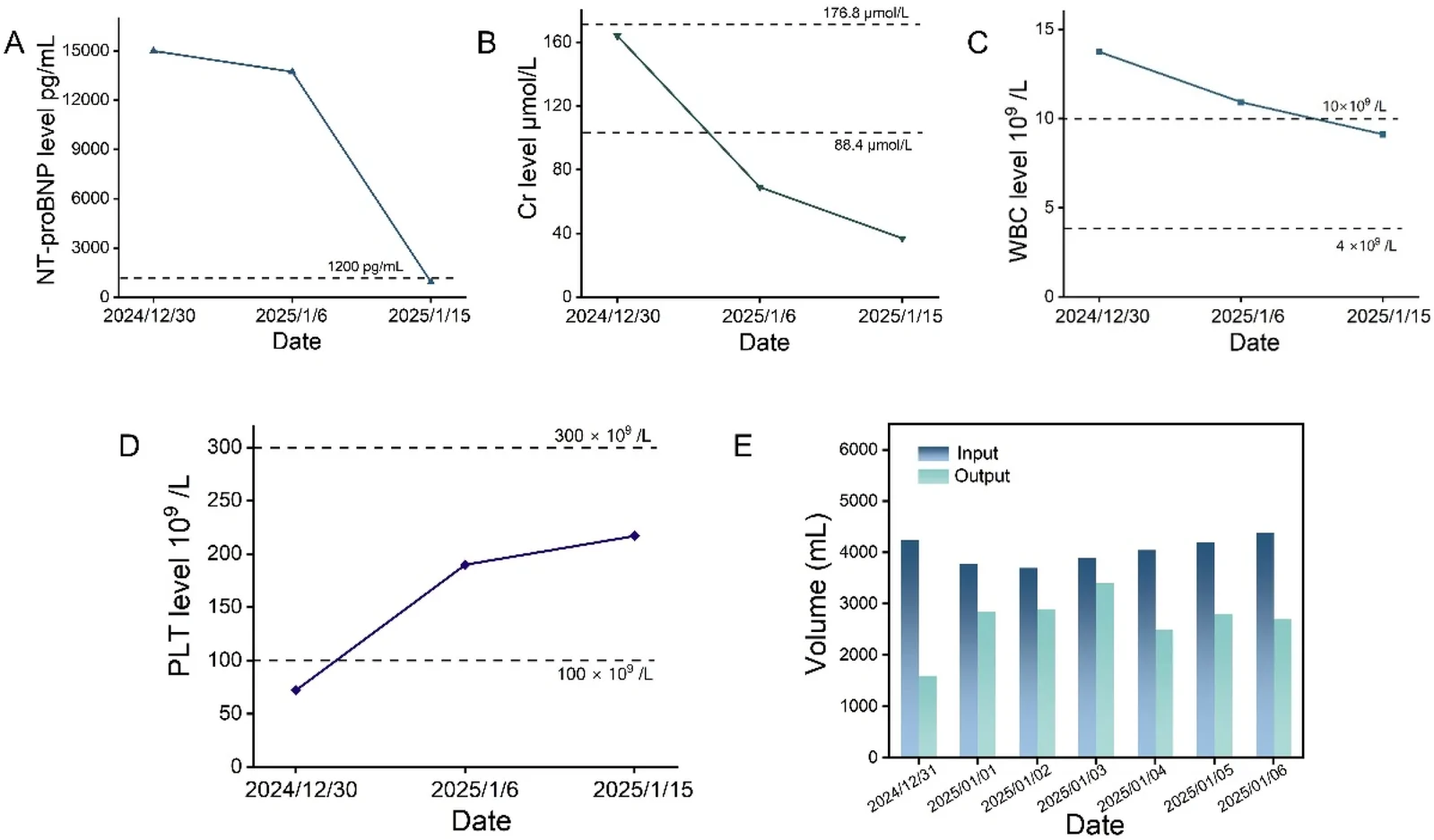
**(A)** NT-proBNP (log scale), **(B)** creatinine, **(C)** white blood cell count, **(D)** platelet count (admission and follow-up). **(E)** Daily fluid balance during the first 7 hospital days (Dec 31 – Jan 7). Sustained negative fluid balance was achieved in the initial 48 h of treatment, verified by hourly urine monitoring. Daily body weights, blood pressure trajectories, and objective ultrasound/BIA volume assessment data were not recorded–these limit interpretation of volume status dynamics.

Volume management: IV fluids (saline/glucose) at approximately 35 mL/kg/day initial target, using the same physiological rationale described in Case 1 (Holliday-Segar derivation, adjusted clinically). Daily fluid balance was recorded. Hourly urine output was tracked for the initial 48 h; target urine output >150 mL/h was sustained after low-dose diuretic plus rhBNP intervention, confirming effective early decongestion (Figure 2E). Daily body weights and precise oral intake were not consistently recorded; therefore, objective assessment of volume status is lacking.

#### Antimicrobial therapy: meropenem

Heart failure management: Low-dose furosemide (20-40 mg/day) + rhBNP (0.01 μg·kg^−1^·min^−1^for 72 h). After hemodynamic stabilization, sequentially started dapagliflozin 10 mg, spironolactone 20 mg, sacubitril/valsartan (50 mg bid titrated up), metoprolol (23.75-47.5 mg/day). Atrial fibrillation rate controlled with beta-blocker, digoxin.

#### Outcome

Infection markers resolved; platelets normalized. NT-proBNP 365 pg/mL at day 15 post-discharge. Creatinine decreased to 46 μmol/L. Discharge LVEF 56%. During outpatient follow-up at our hospital, the patient has remained clinically stable with no re-hospitalization for heart failure. Her current medication regimen includes sacubitril/valsartan 200 mg once daily, metoprolol succinate 47.5 mg once daily, rivaroxaban 15 mg once daily (for atrial fibrillation), and Ginkgo biloba extract 0.25 g three times daily.

## Discussion

This article reports two patients with acute decompensated heart failure who, as part of a comprehensive treatment package including GDMT, antibiotics, and supportive care, received a physiology-guided maintenance fluid strategy. Both patients improved, but this case series cannot determine whether the fluid strategy contributed independently. The following discussion is therefore hypothesis-generating.

## Pathophysiological basis and its limitations

The traditional approach to volume management in heart failure is based on the assumption that all patients experience volume overload. However, the clinical presentation of acute heart failure is heterogeneous. In Case 1, gastrointestinal congestion led to reduced oral intake, resulting in a state of relative hypovolemia manifested as prerenal azotemia. Continuing strict fluid restriction and intensifying diuresis would further reduce renal perfusion, activate the neurohormonal system, and potentially exacerbate heart failure. This represents prerenal kidney injury caused by excessive diuresis. Restoring effective circulating blood volume through cautious fluid replacement may have contributed to interrupting this vicious cycle, resulting in rapid improvement in renal function and a decrease in NT-proBNP, without the need for intensive diuresis. However, we acknowledge that concurrent GDMT and supportive care were major confounders, and improvement cannot be attributed solely to the fluid strategy.

### Case 1 as a scenario of relative hypovolemia, not a general heart failure paradigm

In Case 1, the dominant acute issue was likely dehydration or inadequate intake superimposed on chronic heart failure. Strict fluid restriction would have been harmful. However, this case primarily illustrates the importance of correcting prerenal azotemia rather than supporting liberal fluid administration in typical volume-overloaded ADHF. We explicitly caution against extrapolating this approach to patients without clear hypovolemic features.

### Case 2 and the confounding effect of multimodal therapy

The management of Case 2 was more complex. Sepsis caused peripheral vasodilation, capillary leakage, and increased metabolic demands, leading to relative intravascular volume depletion against a background of systemic congestion. The aggressive fluid resuscitation (30 mL/kg) recommended by sepsis guidelines aims to restore tissue perfusion but is prone to inducing pulmonary edema in patients with poor cardiac reserve; conversely, strict fluid restriction may lead to inadequate organ perfusion and exacerbate septic shock. In this case, a more conservative maintenance fluid approach was used. However, recovery may be explained primarily by source control, antibiotics, resolution of sepsis-induced vasodilation, and GDMT initiation. The fluid strategy was only one component of a bundle. We cannot rule out that standard sepsis resuscitation (30 mL/kg) might have produced similar or better outcomes. Therefore, we present this case only to suggest that a more conservative fluid approach may not be harmful in selected septic HF patients; it does not demonstrate superiority.

### Consistency with contemporary evidence

These observations align with recent trends in the field of fluid management for heart failure. Multiple studies have failed to demonstrate that strict fluid restriction benefits patients with heart failure (3–5) The 2024 ESC Heart Failure Society Consensus Statement explicitly states that routine fluid restriction is not currently supported in patients with acute or chronic heart failure and may be harmful (6). The recently published FRESH-UP randomized controlled trial (2025) challenged the traditional approach to fluid restriction in chronic heart failure (8), finding that liberal fluid intake guided by thirst did not increase adverse events and improved patients’ quality of life (8). Importantly, the FRESH-UP trial enrolled patients with chronic stable heart failure, not acute decompensated heart failure. Its findings suggest that routine fluid restriction may not be beneficial even in stable outpatients, but direct application to acute HF is not valid. We have clarified this distinction. These studies suggest that volume management in heart failure should shift from restriction to optimization, with goals tailored to individual patient characteristics.

## Clinical implications

This report suggests that volume management in acute heart failure requires clinical judgment rather than uniform fluid restriction. Signs of congestion (wet rales, edema, jugular vein distension) and indicators of effective circulating volume (blood pressure, renal function, urea-to-creatinine ratio) must be comprehensively considered to identify potential hypovolemic factors (inadequate intake, infection, high-output state). Fluid management goals should be dynamically adjusted based on physiological requirements (approximately 30–35 mL/kg/day) rather than fixed limits. Per hourly urine output surveillance within the first 24–48 h is strongly recommended by ESC heart failure association position statements and institutional inpatient guidelines to guide decongestive therapy and timely recognize insufficient diuretic response6–7, which we implemented in both cases and presented via daily fluid balance figures.

More importantly, the core of volume management lies in creating stable hemodynamic conditions to initiate and optimize GDMT for improved prognosis. Both patients successfully maintained or initiated GDMT early, suggesting the feasibility. We do not propose 35 mL/kg/day as a standard target for acute heart failure. Rather, we suggest that when hypovolemic factors are present, a non-restrictive maintenance approach guided by frequent reassessment may be considered as an alternative to routine fluid restriction. Individualization is key.

## Limitations

This case series has the following major limitations, many of which are inherent to retrospective case reports and cannot be corrected:

1. Causality cannot be established. Multiple interventions (GDMT, antibiotics, arrhythmia treatment) were initiated simultaneously. The independent effect of the fluid strategy is unknown.
2. Core limitation: Absence of objective, quantitative volume assessment modalities validated by prior clinical studies1–3. Without inferior vena cava ultrasound collapsibility measurement, lung ultrasound congestion scoring, VExUS protocol, bioelectrical impedance analysis (BIA), central venous pressure monitoring, or serial lactate detection, our volume evaluation entirely relied on subjective clinical signs (skin turgor, oral mucosa moisture, lung crackles, jugular venous pressure) and static laboratory ratios (urea/creatinine, serum creatinine). Daily body weight recording was incomplete. Previous prospective trials have proven that BIA and IVC ultrasound provide complementary, reproducible data to distinguish intravascular hypovolemia from interstitial fluid overload in heart failure patients, and improve the accuracy of volume management decisions1–3. The lack of these objective tools greatly reduces the reproducibility and external validity of our physiological-guided fluid protocol. Future prospective validating trials must incorporate BIA and IVC ultrasound as standardized baseline assessments.
3. The 35 mL/kg/day target lacks evidence. This value was derived from general maintenance fluid calculations, not from heart failure-specific data. Whether 25 or 40 mL/kg/day would yield similar results is unknown.
4. Oral intake was estimated, not precisely measured.
5. Short follow-up and small sample size. Results are not generalizable.
6. Case 1 may primarily represent correction of dehydration rather than a novel HF management paradigm.

## Conclusion

These two cases are hypothesis-generating only. For selected patients with acute heart failure and possible hypovolemic factors (poor intake, sepsis-related losses), a physiology-guided maintenance fluid strategy may be considered as an alternative to strict restriction, but its efficacy and safety remain unproven. Volume management should be individualized, and prospective studies with objective hemodynamic monitoring are needed before any recommendation can be made.

## Data Availability

The original contributions presented in the study are included in the article/Supplementary Material, further inquiries can be directed to the corresponding authors.
